# Targeting ethical considerations tied to image-based mobile health diagnostic support specific to clinicians in low-resource settings: the Brocher proposition

**DOI:** 10.1080/16549716.2019.1666695

**Published:** 2019-09-18

**Authors:** L. Laflamme, J. Chipps, H. Fangerau, N. Juth, F. Légaré, H. R. Sawe, L. Wallis

**Affiliations:** a Department of Public Health Sciences, Global Health, Karolinska Institutet, Widerströmska Huset, Stockholm, Sweden; b Institute for Social and Health Sciences, University of South Africa, Johannesburg, South Africa; c School of Nursing, University of the Western Cape, Belville, South Africa; d Department of the History, Philosophy and Ethics of Medicine, Medical Faculty, Heinrich-Heine-University Duesseldorf, Duesseldorf, Germany; e Department of Learning, Informatics, Management, and Ethics, Karolinska Institutet, Widerströmska Huset, Stockholm, Sweden; f Centre de recherche sur les soins et services de première ligne de l’Université Laval, CIUSSS de la Capitale-Nationale, Quebec City, Quebec, Canada; g Department of Family Medicine and Emergency Medicine, Faculty of Medicine, Université Laval, Quebec City, Quebec, Canada; h Emergency Medicine Department, School of Medicine, Muhimbili University of Health and Allied Sciences, Dar es salaam, Tanzania; i Division of Emergency Medicine, Faculty of Medicine and Health Sciences, Stellenbosch University, Bellville, South Africa; j Division of Emergency Medicine, Faculty of Health Sciences, University of Cape Town, Cape Town, South Africa

**Keywords:** mHealth, ethics, diagnostic support, autonomy, patient safety, low resource settings

## Abstract

**Background**: mHealth applications assist workflow, help move towards equitable access to care, and facilitate care delivery. They have great potential to impact care in low-resource countries, but have significant ethical concerns pertaining to patient autonomy, safety, and justice.

**Objective**: To achieve consensus among stakeholders on how to address concerns pertaining to autonomy, safety, and justice among mHealth developers and users in low-resource settings, in particular for the application of image-based consultation for diagnostic support.

**Methods**: A consensus approach was taken during a three-day workshop using a purposive sample of global mHealth stakeholders (n = 27) professionally and geographically spread. Throughout a series of introductory talks, group brainstorming, plenary reviews, and synthesis by the moderators, lists of actions were generated that address the concerns engendered by mHealth applications on autonomy, justice and safety, taking into account the development, implementation, and scale-up phases of an mHealth application lifecycle.

**Results**: Several types of actions were recommended; key ones among them included building in risk mitigation measures from the development stage, establishing inclusive consultation processes, using open sources platform whenever possible, training all clinical users, and bearing in mind that the gold standard of care is face-to-face consultation with the patient. Recommendations of patient, community and health system participation and of governance were identified as cutting across the mHealth lifecycle.

**Conclusion**: Priorities agreed-upon at the meeting echo those put forward concerning other domains and locations of application of mHealth. Those more forcefully articulated are the need to adopt and maintain participatory processes as well as promoting self-governance. They are expected to cut across the mHealth lifecycle and are prerequisites to the safeguard of autonomy, safety and justice.

## Background

The conjunction of smartphones’ increasing versatility and wireless networks’ widening coverage has sparked wide-ranging digital approaches in health care [1–3]. Mobile health (mHealth) applications receive support from health care professionals, for assisting the workflow, from institutions concerned with reducing global poverty, for prospects of equitable access to care, and from promotors of patient empowerment, for facilitating the process [2,4–6]. Low-resource countries, especially in Africa, are frequent environments for clinician-to-clinician applications dedicated to diagnostic and management assistance, several of which are non-medical like WhatsApp ®. Despite noteworthy individual and societal potential benefits addressing the human right for health care [7], those applications foster tangible ethical concerns pertaining to other human rights like patient autonomy, safety, and justice [8–15]. At the forefront lie the loss of privacy inherent in how information is handled, and threats to patient safety emerging from weaknesses in the quality of the digital information inherent in the iterative development process of mHealth apps [1,6,14,16]. Errors as well as mischievous additions are a main concern, from silent ones, built-in during the app development [17,18], to those due to the clinical users’ limited qualifications [18,19], or the unstandardized and unsupervised environments of use [6,16,19].

Image-based consultation can assist in decision making relative to triage, treatment, and disposition, and in following up treatment progression [20]. Sharing and receiving images through social media is part of the clinical culture of communicating [21] and, often, a number-one choice [22] for both primary and secondary purposes (i.e. patient care versus e.g. medical research and education). Instant messaging has spread as a practice and support physician-to-physician systems in many parts of the world across all resource levels [23,24], but it presents particular ethical concerns [14]. As for multimedia information, clinical images are transmitted or published without having either the clinician or the patient fully aware of the breach in individual privacy that may occur [20,25]. Also, using clinical images in tandem with social media is not the same as telemedicine [20], which is a real-time event constructed and configured in a way that there are built-in safeguards for protecting the distribution and storage of the clinical records that social media does not provide. Further, medical images are no longer solely taken by professional clinical photographers [14,20] but, rather, by healthcare providers that may lack specific qualification and training [20].

Currently, recommendations to mitigate concerns pertaining to the ethical principles of autonomy, safety and justice are available in the scientific literature and professional guidelines (e.g. medicine, nursing or engineering) [1,26]. Those compilations are seldom disaggregated according to the stages of the lifecycle of mHealth applications (development, evaluation of efficacy and effectiveness, implementation, and scale up) [27], their acceptance across a range of stakeholders is uncertain, and their applicability to low-resource settings has received limited attention. This study aims to fill those knowledge gaps by investigating what stakeholders from different settings and backgrounds agree upon and prioritize to tackle autonomy, safety, and justice concerns when using image-based mHealth applications in resource-poor settings.

## Methods

In January 2019, a three-day workshop was held at the Brocher Foundation [28] co-organized by Swedish and South African researchers, all involved in point-of-care mHealth studies in low-resource settings. The following ideas guided the preparation for the event:

- Assess the current state of knowledge on potential ethical concerns.

- Look at solutions one ethical principle at a time and in consideration of key phases of mHealth application lifecycle.
- Stimulate individual reflections both before (providing background documents and checklists [1,26] and during the meeting (integrating brief state-of-the-art presentations).
- Stimulate open discussions where all can have a say (e.g. through interactive small group sessions and wrap-up plenaries).

The participants consisted of a purposive sample of 27 people, 12 women and 15 men, identified through prior involvement in similar events, expert reputation, or on the basis is of their published work in the field. Lay public and patients were not invited, due to space considerations (given the capacity of the venue, there is a maximum of 30 for a Brocher event). We invited 35 participants, but not all were able to attend. Participants represented their own views rather than those of their organisation. All were invited by email, using a standardized letter. They were employed in governmental agencies, public and private organisations or universities, and were geographically and professionally spread, as shown in Table 1.

**Table 1.** Distribution of the workshop participants by sector of activity and country.

	Country				
Sector	South Africa	Africa Other^$^	Sweden	Europe Other*	Canada, North America
Clinical	2	2			2
Research	1	1	4	3	2
Medical ethics	1		1		
Health policy		2		4	
Developers/business	1				1

*Finland, Germany, Switzerland ^$^Mali, Tanzania, Uganda.

**Figure 1.**
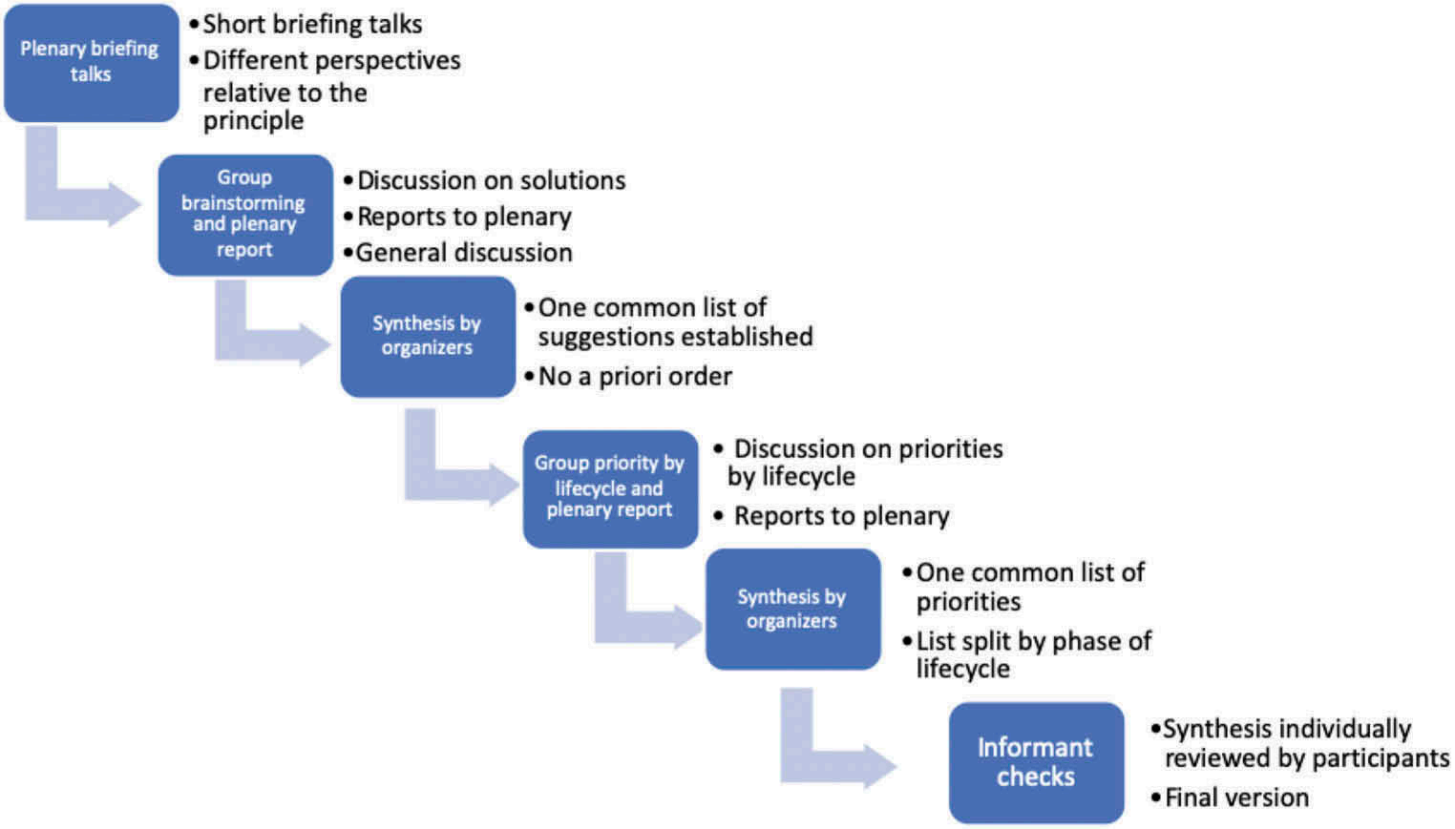
Overview of the process followed during the workshop to guide the discussions and generate agreed-upon views on how to tackle issues pertaining to each ethical principle covered (autonomy, safety, and justice).

To determine the most consensual and highly prioritised actions that can be taken to safeguard patients’ and other users’ autonomy, safety, and justice, and to organise them around critical phases of applications’ lifecycle [27], we proceeded as follows: from the start, participants were divided into groups with a geographic, gender and speciality mix, and each group had an assigned chair. Thematic sessions unfolded, starting with reflections around patient issues, followed by those around clinical users and health systems. As indicated in Figure 1, for any given ethical principle, participants were asked to have in mind the whole lifecycle of mHealth apps, but they were not forced to organise their discussions around it. Likewise, they were asked to consider above all, the perspective of low-resource settings.

As indicated in Figure 1, throughout a series of introductory talks, group brainstorming, plenary reviews, and synthesis by the moderators, a list of solutions susceptible to help tackle the ethical threats engendered by mHealth applications at the development phase, implementation or scale-up eventually materialised. Group discussions were captured by note taking at each table and by two rapporteurs. At the end of each session, and at the end of days 1 and 2, leaders of the initiative met and content-analysed this material and highlighted priorities that were agreed upon by the participants.

The participants took part in the workshop on a voluntary basis and participation did not require the signature of an informed consent. Approval from an ethics board was not required.

## Results

### Autonomy

The 13 actions related to the principle of autonomy and respect of person concerned, above all, ensuring that patient data is safely dealt with. This should be addressed from building-in mechanisms in the development process to establishing clear rules at time of scale-up (see Table 2). Using open source platforms was put forward, and so was the user-friendliness of the applications developed, and even the engagement of all stakeholders throughout the whole lifecycle. At the implementation and scale-up phases, informed consent (with all building components), and understanding and ownership (e.g. through self-governance) stand out as necessary aspects, alongside follow up and maintenance of the whole system.

**Table 2.** Synthesis of the priorities raised by the workshop participants related to respect of person and patient autonomy, split into the main lifecycle phases of mHealth applications.

Development	Implementation	Scale up/Follow up
Incorporate data security and patient privacy into the design process	Prioritise and always strive for consent that is informed, face-to-face, and individual	Establish clear rules of usage for informal mHealth systems that are consistent with the existing ethical and regulatory framework
Have a regulatory framework in place that safeguards data protection	Make use of existing local ethical guidelines	Ensure that continuous maintenance and updates of the system are in place
Use an open source data collection platform	Raise mHealth awareness among all stakeholders	Safeguard and maintain open source platforms
Build in secured mechanisms to facilitate efficient exchange of data into the existing health record system	Avoid perverse incentives that interfere with providers’ decision to use the system	
	Foster local governance of digital applications within the country’s health care system	
Engage all stakeholders throughout the whole process		

### Safety

Table 3 presents the 14 solutions to mitigate safety concerns. What stands out is the consensus around the need to build in safety promoting mechanisms from the start (and ensuring they are maintained from then on), and the need for evidence-based processes and for clear and well-understood rules, procedures and standards of care. Additional points that come across from the implementation phase are the imperative need for informed consent, the alignment of safety promoting rules to already-existing ethical guidelines, and the empowerment of clinical users in their capacity to use the system. For implementation and scale-up, understanding and ownership (e.g. through self-governance) received priority, alongside follow up and maintenance. The necessary engagement of all stakeholders and local governance throughout the whole lifecycle were both forcefully stressed.

**Table 3.** Synthesis of the priorities raised by the workshop participants related to patient safety, split into the main lifecycle phases of mHealth applications.

Development	Implementation	Scale up/Follow up
Proceed to development using a robust scientific process	Proceed to implementation following the principles of implementation science	Ensure ongoing, effective phone stewardship
Incorporate data security and patient privacy into the design process	Ensure mHealth literacy: all clinical users are trained in adequate device stewardship	Assure compliance of the system with a locally-agreed safety level
Incorporate a robust user authentication system into the design	Familiarise all clinical users with the overall process	With systems that employ artificial intelligence, ensure processes are in place to encourage the maintenance of clinical staff diagnostic skills
Make explicit the permissible (‘good enough’) standard-of-care being targeted, and ensure it has been set in a fair and transparent manner	Define clearly standards of clinical care that are desirable, permissible, and forbidden	Ensure continuous quality improvement mechanisms are in place
Avoid conflicts of interest or perverse incentives for developers		
Engage all stakeholders throughout the whole process		

### Justice

Table 4 presents the 13 solutions targeting justice, of which several rests on the application development phase. Justice was considered in a broad sense, and included the equal right of access to mHealth, and also that of being involved in decisions pertaining to what health issues should receive priority in developing mHealth solutions.

**Table 4.** Synthesis of the priorities raised by the workshop participants related to justice, split into the main lifecycle phases of mHealth applications.

Development	Implementation	Scale up/Follow up
Assure the end product is accessible (i.e. affordable and robust)	Include a robust maintenance plan in the implementation strategy	Identify and prioritise target groups in a fair and transparent manner
Implement an open source data collection platform	Safeguard the engagement of all stakeholders throughout the implementation process	Ensure the system is equally accessible to the entire target group(s)
Incorporate interoperability of the final product into the existing health care system from inception		Assess the system’s ability to reach out to all segments of the population/population groups
Make explicit the permissible (‘good enough’) standard of care being targeted and ensure it has been set in a fair and transparent manner		
Engage all stakeholders throughout the whole process		
Develop apps in a transparent manner		
Encourage ‘bottom up’, locally-relevant development that aligns with local health priorities		
Balance use of existing resources with the need to drive innovation		

## Discussion

The priorities agreed upon disentangle interventions needed at specific lifecycle phases, some of which pertain to more than one phase or touch upon more than one ethical principle.

### Similar requirements albeit a new context

In spite of image-based being the specific field of application of mHealth covered at the meeting, many priorities put forward resemble those highlighted for mHealth applications in broader fields of utilization or in better well-off settings [1,3,8–15]. Sovereignty, participation and non-discrimination cut across all phases, reflecting fairness, respect, equality, dignity and autonomy as a human rights-based approach to health care [29].

Having the prevention mechanisms built in from the start is one example, and it faces a similar barrier: mHealth app creators are not covered by guidelines like the Health Information Portability and Privacy Act (HIPAA) or the International Medical Informatics Association (IMIA) code, and lack incentive to provide robust information security mechanisms. Also echoing other fields of utilization of mHealth are the need to allow for monitoring and follow up, and thereby ensure the responsiveness of a system to both users’ needs and changing environments. This would, in turn, ensure privacy is maintained, patients’ rights are preserved, and diversity of stakeholders that need to be involved – from within and outside the health sector – is promoted. One final similarity is that the instrumentality of mHealth is emphasized: the gold standard of care must remain a face-to-face consultation with the patient. Point-of-care workers must be provided with clear information – and trained accordingly – as regards what the acceptable standard of care in the local health system context is. Using mHealth solutions should not be an excuse for substandard care, especially since it is likely to target patients in resource-poor settings, i.e. those who are in many respects already worse off. This is both a safety and a justice issue. Both prioritarian and egalitarian considerations of justice imply that such groups have stronger entitlements to receive support rather than weaker [30].

### Informed consent and governance at the forefront

The stakeholders emphasized that patient authorization must remain a prerequisite to any mHealth intervention, something that is particularly critical in image-based consultation [14,20,31–33]. Data – more or better – must not take precedence over patient [9], and uncertainties experienced by point-of-care workers [15,18,34,35], in relation with patients and other system users, must be dealt with. Instrumental to this are the rights for the users to be consulted at all phases and to be adequately trained. Beyond individual users, the stakeholders also insisted on the necessary involvement of local communities at all phases and on the right to governance, two points that were not as much emphasized in earlier studies. At the workshop, the ‘local’ or even ‘regional’ character of medical innovations was stressed in many ways, from requirements to meet local priorities and engage local stakeholders to others of using locally-derived or agreed upon clinical standards and ethical principles.

### Informed consent and justice – challenges ahead

The actual feasibility – and ultimately even desirability – of informed consent was debated during the meeting. The discussions were not straightforward – including the level(s) at which it must be sought (individual vs community), and who between the community and the individual patient has the final word. The notion of informed consent came across as a complex one; tensions may arise between whose consent is required (for both primary and secondary use of information), conflict between community and individual consent, and the degree to which patients can be expected to actively engage.

As for justice, there are aspects that need consideration at both group and individual levels. In the former case, the fact that many mHealth solutions are meant to reach out to and improve the situation of (groups of) patients in resource-poor settings, egalitarian justice is promoted. But group-level tensions persist if, for instance, only some health conditions are targeted by mHealth whereas others are ignored. How such tensions can be dealt with remains to be determined. At the meeting, the discussions were concentrated around distributive principles of justice, like prioritarian (emphasising the need to reduce the burden of those worse-off) and egalitarian (reducing relative inequalities between groups and individuals) ones, but not as much with other considerations like that of reciprocity or even responsibility [36]. As a consequence, whether and in what way aspects of the like should be built into future guidelines or revisions of existent ones is uncertain. However, there was broad consensus in favour of the egalitarian standpoint favouring equal accessibility to the whole target group(s). Failure to achieve this gives rise to legitimate justice-based complaints from the disadvantaged individuals within these groups.

### Strengths and limitations

The solution-oriented approach that was followed materialised in prioritized actions that were agreed upon among knowledgeable and experienced stakeholders. In events of the like (see other examples in [35,37], high-level governmental and business people are typically under-represented [35] and their views are unweighted [29]. But there are good prospects of collaborations around mHealth education, delivery and integration [37] not least in sub-Saharan Africa [38].

The final wording of the suggestions was determined by the organisers, after participant checks. The principle- and phase-specific lists were made longer than shorter: when two or more solutions were interrelated but not fully overlapping, they were all kept. This is very apparent for the principle of justice, in the development phase.

Although the workshop aimed to address ethical challenges affecting all mHealth users (patients, frontline and experts) some may have been overshadowed during the meetings. In addition, due to space considerations, we focussed on groups other than patients and public: as can be seen from the results, the need for patients’ voices was strongly emphasised in all three ethical areas, meaning that this group must be well represented, not least at any such future events.

## Conclusions

The set of propositions resulting from the Brocher meeting shows that many actions can be taken to safeguard that crucial ethical principles as autonomy, safety and justice be respected when using image-based mHealth. There is overwhelming consensus regarding the need to foresee and build-in ethically-oriented solutions from the development stage, and to follow an evidence-based and inclusive process throughout the lifecycle of any application. Many see it as essential that self-governance is a mantra during the whole lifecycle of mHealth, on all levels and in all its dimensions – individual, community, heath system, and nation.

## Paper context

Image-based mHealth can significantly reduce the burden of disease and its unequal social distribution and impact. There are significant ethical concerns in its usage, especially in low resource settings. Stakeholders gathered at a three-day workshop helped to elaborate these issues, focusing on issues relative to front-line users and organizational and technical environments. Ethical challenges and their potential solutions were addressed in terms of autonomy, safety, and justice
